# A Study of a New Technique of the CT Scan View and Disease Classification Protocol Based on Level Challenges in Cases of Coronavirus Disease

**DOI:** 10.1155/2021/5554408

**Published:** 2021-03-18

**Authors:** Ahmed B. Salem Salamh, Abdulrauf A. Salamah, Halil Ibrahim Akyüz

**Affiliations:** ^1^Institute of Science, Material Science and Engineering, Kastamonu University, Kuzey Kent /P.O. Box, 37150, Kastamonu, Kastamonu, Turkey; ^2^Tripoli Central Hospital, P.O. Box 15528, Tripoli, Libya; ^3^Computer and Teaching Technologies Education, Kastamonu University, Kuzey Kent /P.O. Box, 37150, Kastamonu, Kastamonu, Turkey

## Abstract

The chest Computer Tomography (CT scan) is used in the diagnosis of coronavirus disease 2019 (COVID-19) and is an important complement to the Reverse Transcription Polymerase Chain Reaction (RT-PCR) test. The paper aims to improve the radiological diagnosis in the case of coronavirus disease COVID-19 pneumonia on forms of noninvasive approaches with conventional and high-resolution computer tomography (HRCT) scan images upon chest CT images of patients confirmed with mild to severe findings. The preliminary study is to compare the radiological findings of COVID-19 pneumonia in conventional chest CT images with images processed by a new tool and reviewed by expert radiologists. The researchers used a new filter called Golden Key Tool (GK-Tool) which has confirmed the improvement in the quality and diagnostic efficacy of images acquired using our modified images. Further, Convolution Neural Networks (CNNs) architecture called VGG face was used to classify chest CT images. The classification has been performed by using VGG face on various datasets which are considered as a protocol to diagnose COVID-19, Non-COVID-19 (other lung diseases), and normal cases (no findings on chest CT). Accordingly, the performance evaluation of the GK-Tool was fairly good as shown in the first set of results, where 80–95% of participants show a good to excellent assessment of the new images view in the case of COVID-19 patients. The results, in general, illustrate good recognition rates in the diagnosis and, therefore, would be significantly higher in normal cases with COVID-19. These results could reduce the radiologist's workload burden and play a major role in the decision-making process.

## 1. Introduction

**Figure 1:**
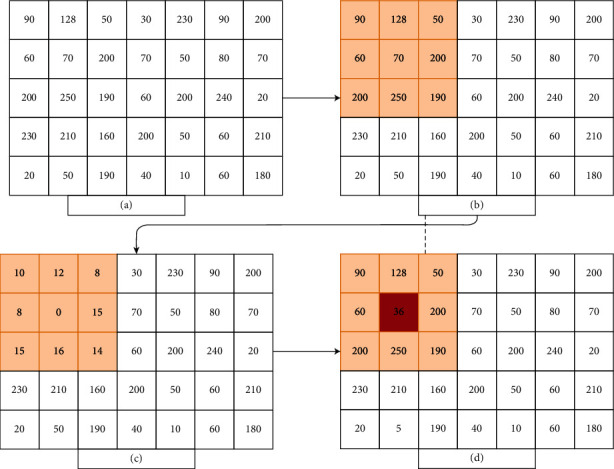
Demonstration of the steps to compute the square root value. The result would be varying based of *P* value and each value of square root must be rounded off. (a) Example of the original example of the image, (b) The determination of 3 neighbors, (c) The square root of 3 neighbors of center value, and (d) The final equation result value with *P*=5.

In the first half of this year, on March 13, 2020, the World Health Organization (WHO) officially notified that the novel coronavirus disease (COVID-19) had become a global pandemic. COVID-19 is caused by severe acute respiratory syndrome coronavirus 2 (SARS-CoV-2) depicting a potentially fatal disease. [1]. Many previous studies have reported that the CT imaging features and correlation of chest computed tomography (CT) and reverse transcription polymerase chain reaction (RT-PCR) testing showed infections with SARS-CoV-2 [2–4]. The high false-negative rates and time-consuming RT-PCR affect the efficacy of this test [5, 6]. The asymptomatic cases of COVID-19 pneumonia CT scan images have definite characteristics and have important value in screening and detecting patients with COVID-19 pneumonia, especially in the highly suspicious cases with negative PCR testing. [7, 8]. Another diagnostic method to diagnosis and treatment guideline for COVID-19 pneumonia, issued by the National Health Commission of The People's Republic of China is the CT, especially the high-resolution CT (HRCT) [9]. The CT examination of patients with COVID-19 pneumonia shows mixed and various patterns with both lung parenchyma and interstitial involvement. Represented with ground-glass opacity (GGO) with different manifestations which could be a solitary lesion at early-phase disease, the signs of aggravation and repair are seen in the advanced phase. Lesions presented with characteristic multifocal areas predominantly distributed in the middle and lower lung regions and in the posterior lung area. [10]. The most common chest findings of COVID-19 in CT images are multiple GGO, consolidation, crazy-paving, and interlobular septal thickening in both lungs, which are mostly distributed at subpleural locations in the lower lobes but the findings are nonspecific and can be found in other illness, including cancer [11]. There are significant correlations between the degree of pulmonary inflammation and the main clinical symptoms and laboratory results. In the emerging global health emergency, the computed tomography plays a major role in the diagnosis and evaluation [12, 13]. Usually, the chest CT abnormalities significantly increased in number up to 10 weeks (the peak at approximately 10 days). Subsequently, there were a short plateau phase and a gradual decrease in abnormalities with time [14]. There is obvious evidence that fatigue, particularly after long shift duty, is related to serious medical errors and can increase the risk of missing the image findings, especially during and pandemic time that complicates the situation and leads to more medical errors. [15, 16]. In general, it is divided into two main subtypes: the first one is physical and the second is mental fatigue. Especially in outbreak time, it is noted that physical fatigue comes first, which leads to deterioration in the muscle ability to create or sustain forces, which happens due to difficulties in controlling and coordinating muscles. Mental fatigue is a reduction in the ability to perform mental tasks and responses [17]. In general, newer techniques can potentially facilitate radiology department workflow, increase radiologist accuracy, improve detection of findings, reduce the possibility of medical error, and enhance patient health care [18]. Moreover, the outbreak likely persists for a foreseeable period as well with peaks. For immediate preparedness, the radiology in its ongoing battle against COVID-19 must reconsider and readjust the workflows and leverage new technologies and applications [19]. Recent modern trends show increased and rapid use of machine learning (ML) in the medical field, including computer-aided diagnosis (CAD), radio-mics, and radiology department [20]. In the last century, the first class of Neural Networks (NNs), called Convolutional Neural Network (CNN) was reported which showed enormous potential in machine vision (MV) related tasks [21]. The CNN designs are based on the architecture that performs traffic sign classification related tasks with a good recognition rate. This method uses a deep CNN which has been widely used for image classification and trained to classify faces using a VGG face (Deep Face) Figure 1 [8, 22]. The CNN “deep learning,” in medical imaging is an explosively growing, promising field [23, 24].

## 2. Patients and Methods

### 2.1. Patients

**Figure 2:**
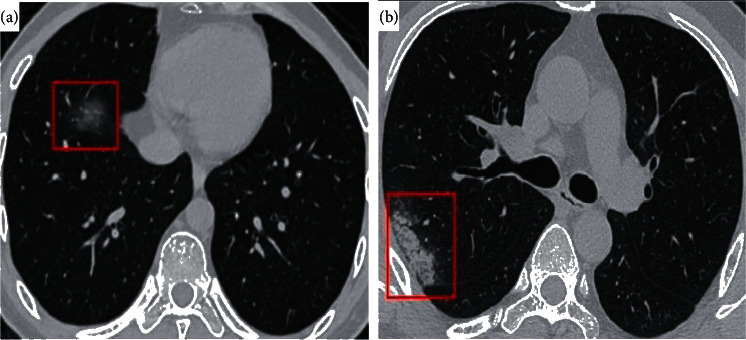
(a) Chest CT scan image shows a pure ground-glass opacity (GGO) in the right lower lobe (red frame). (b) Consolidation in the right lobe subpleural area (red frame).

Now we know that the COVID-19 chest CT view shows a pattern of ground-glass opacity in the lung leading to “consolidation” in advanced stages of the diseases. As seen in Figure 2. CT results become more widespread for a longer duration after the onset of symptoms, including consolidation, bilateral and peripheral disease, and overall lung involvement, linear opacities, “crazy-paving” pattern, and the “reverse halo” sign [25]. The data utility of CT views in the case of COVID-19 has been confirmed by a senior radiologist in Tongji Hospital, Wuhan, China, who had performed the diagnosis and treatment on a large number of COVID-19 patients during the outbreak of this disease between January and April [16, 26].

### 2.2. CT Images Acquisition and Preprocessing

The data of original CT images quality degradation during different utility and their negative impacts included Hounsfield unit (HU), any values lost, the number of bits per pixel, the resolutions reducing in comparison with original CT images, and putting into papers and about choosing only a few key slices to detect the diagnosis. The researchers consulted the aforementioned radiologist from China Tongji Hospital about these concerns. The problems posed in these considerations do not substantially affect the accuracy of diagnosis decision-making, according to the radiologist. They confirmed that any basic experienced radiologist will be able to make accurate diagnoses from low-quality CT images. The quality gap between CT images in papers and original CT images may not largely affect the accuracy of diagnosis. Moreover, one read of a single-slice of CT may provide enough clinical information for accurate decision-making. The information about the tomography type, brand, model, and fabrication companies are not available due to the characteristics of the retrospective study.

### 2.3. CT Image Square Root Equation

The main objective from design preprocessing filter to change lung CT image view from conventional view which means black and white to newer ones which can illustrate such improvement in the pattern with a new view. This can help in the major differentiation between the findings in COVID-19 cases. Square root equation (SRE) (1) operator's main idea is as follows: the first step is to convert lung CT image to grayscale. The second step is taking pixel point (*x*_T_, *y*_T_) as the center point of 3–3 windows in an (m-n) image. The third step is applying the square root around the central point pixel value (*x*_T_, *y*_T_) as *g*_*i*_, with the adjacent eight-pixel values being a_0_-a_7_, Figure 2.

Total summation of squaring adjacent eight-pixel values with the subtraction of 255 value with introducing the final feature point replaced by the central point pixel value as shown in the following equation:

(1) $$\begin{array}{c} SRE\;=255\;-\;\sum_{s=0}^{Z-1}\left(\sqrt[{2}]{\left(adjacent\;\left(g_{s}\right.\right)}*\sqrt[{2}]{p}\right)\;, \end{array}$$

where *z* = 8, *p* = 1, 2,……….N : *N* ≤ 16 and *g*_*s*_ three neighbors of center value. The value of *p* is represented from 1 to 16 which introduce various differentiations. Based on this, each value of *p* must be selected to accommodate its applications. The pseudocode for the square root equation is as seen in Algorithm 1.

### 2.4. Deep Learning Model

In the case of image classification, Convolution Neural Network is the most important field in the sense of machine vision. CNN has obtained several achievements in the area of classification, segmentation, and recognition. The instant grayscale image is a 2D array and a color image is a combination of three 2D arrays. The CNNs are designed to operate on data that is in the form of multiple arrays [27].

**Figure 3:**
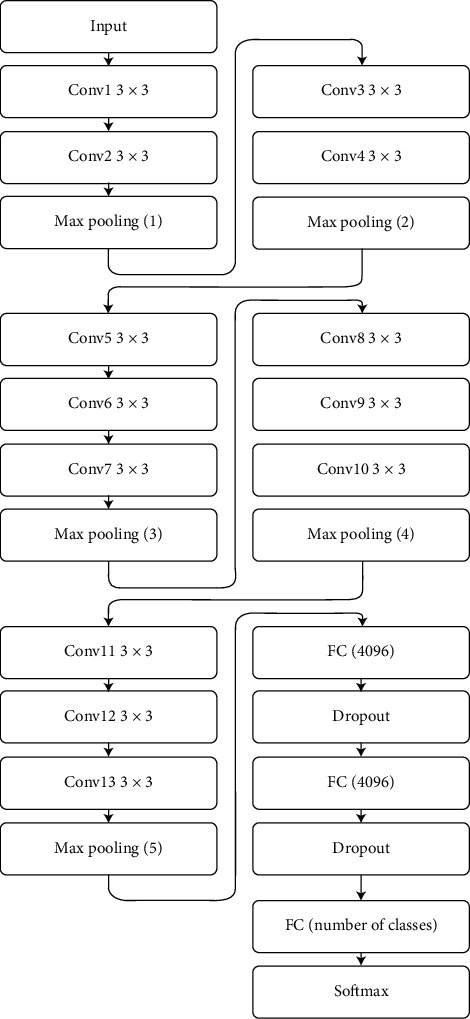
Deep learning VGG face architecture.

The major field of CNN is in medical imaging, particularly for the diagnosis of cancer using image classification based on histopathological images. CNN recently is often used to diagnose images of breast cancer, and findings are contrasted with a network trained on a dataset containing local features descriptors [24, 28].  Figure 3 represents the VGG model of CNN used in this paper presenting in detail the CNN with its configuration in Table 1. The model used in this paper is based on the following steps:

- Pretrained model
- Transfer learning
- Fine-tune the model

### 2.5. Matrices Evaluation and Statistical Analysis

In order to evaluate and compare the performance of the CNNs, the indices were as follows:

(i) Accuracy (Acc):
  (2) $$\begin{array}{c} ACC=\;\frac{TP+TN}{TP+TN+FP+FN\;}\;. \end{array}$$
(ii) Error rate (ERR):
  (3) $$\begin{array}{c} EER=1-ACC\;. \end{array}$$
(iii) Sensitivity or true positive rate (TPR):
  (4) $$\begin{array}{c} TPR=\frac{TP}{TP+FN}. \end{array}$$
(iv) Specificity or true negative rate (TNR):
  (5) $$\begin{array}{c} TNR=\frac{TP}{FP+TN}\;. \end{array}$$
(v) Positive prediction value (PPV):
  (6) $$\begin{array}{c} PPV=\frac{TP}{FP+TP}. \end{array}$$
(vi) False positive rates (FPR):
  (7) $$\begin{array}{c} FPR=1=TNR. \end{array}$$
(vii) F-Measure or F1 score
  (8) $$\begin{array}{c} \text{F}1\_\text{score}=\frac{2TP}{2TP+FP+FN}. \end{array}$$
(viii) Matthews's correlation coefficient (MCC)
  (9) $$\begin{array}{c} MCC=\frac{TP*TN-FP*FN}{\sqrt{\left(TP+FP\right)\left(TP+FN\right)\left(TN+FP\right)\left(TN+FN\right)}}. \end{array}$$

## 3. Results and Discussions

**Figure 4:**
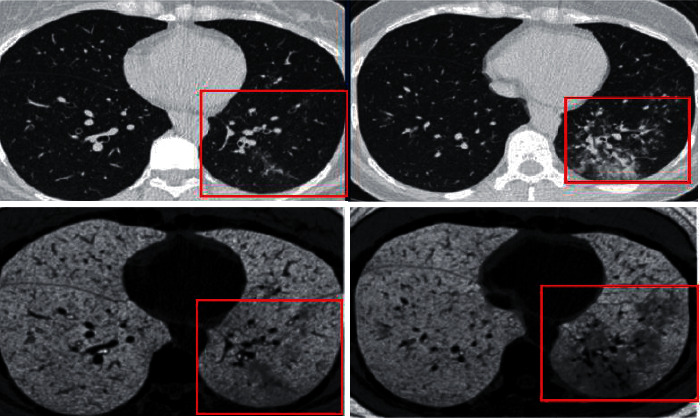
Lung CT images of COVID-19 findings before and after the process. The findings are in (red frame).

The presentation images view in CT scan and the sensitivity in radiology diagnosis and detections can be improved. These improvements further have an effective potential in clinical applications, implementation, and structure. It also enhances the role of radiologists, who indirectly have a strong impact on radiology department outcomes especially during overload works. The diagnosis of COVID-19 by the use of imaging processes called GK-Tool with other conventional CT chest scans (COVID-19 pneumonia) is being compared and assessed by radiologists. In this study, the researchers have used CT images of patients already confirmed with COVID-19. The diagnosis has been made on patients belonging to both genders. The radiologists and experts in this field who reviewed the images have good experience in thoracic imaging for more than five years. All radiologists have reviewed the CT images of COVID-19 patients with defined regions of infections and reviewed the lung CT slices of different images with and without processing as seen in Figure 4. The performance reports were collected based on visual inspection of CT different images slices and views on a given scale from 1 to 5 points. The results illustrate that 80–95% of participants gave “good to excellent” assessment ratings on the images view improvement and the diagnosis sensitivity in the case of COVID-19 patients; refer to Tables 2 and 3. The researchers have considered engaging several radiologists from different hospitals and from different academic backgrounds to get an improved perspective and evaluation.

The critical and important issues for radiologists are to improve the sensitivity in the diagnosis and differentiating of COVID-19 from other typical diseases. The CNN's “deep learning” in medical imaging is rising exponentially thereby providing the detection of several diseases. The technology of using CNN VGG face can be very helpful to improve the performance in the radiology department. Further, it can give a rapid triaging of radiological findings detection and the technique that can potentially facilitate radiology department workflow. However, this paper is constrained to describe technical information and internal processing (data entry, implementation of CNNs, training, and result phases) about deep learning with CNNs by construction which contains different identities based on CT images' findings from our dataset. The four datasets taken as the protocol in order to assist the decision-making for any global pandemic are as mentioned below.

### 3.1. Dataset 1: COVID-19 and Normal Cases

**Figure 5:**
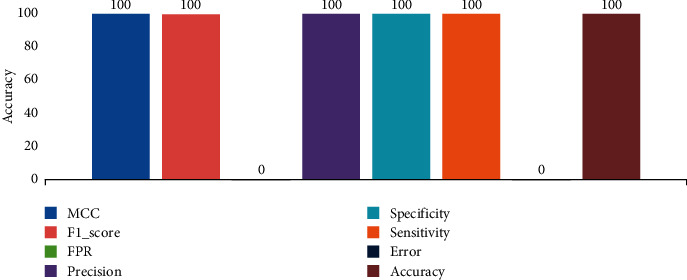
Overall, the values of COVID-19 with normal cases.

Dataset 1 is a comparison between chest CT images of COVID-19 cases with normal cases. The researchers entered 349 CT images with different slices of confirmed cases of COVID-19 and 55 images of normal image cases. The dataset of COVID-19 cases included the early stage, presented with pure GGOs progressive stage; multiple GGOs, consolidations in lesions, crazy-paving pattern and the extensive stage, diffuse exudative lesions and white lung lesions [13, 29]. The evaluation performance of COVID-19 with normal cases achieved an accuracy of 100% for all matrices indicating that the system handles the problem perfectly. Refer to Figure 5.

### 3.2. Dataset 2: COVID-19 with Normal and Non-COVID-19 (20) Cases

**Figure 6:**
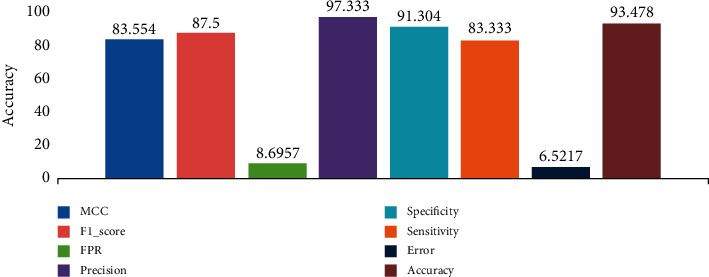
COVID-19 with normal and non-COVID-19 cases.

Dataset 2 shows a comparison between chest CT images of COVID-19 cases with non-COVID-19 cases (other lung diseases that have similar findings in chest CT scan). The sample entered 349 chest CT images with different slices of confirmed cases of COVID-19, 55 images of normal image cases, and 20 images of non-COVID-19 cases. The evaluation matrices show fairly good accuracy 93.478% with an error of 6.5217% as seen in Figure 6. The precision showed the proportion of positive classes truly classified to the total number of positive predicted as 97.33%. F1 score gives the harmonic mean of precision and recalls with 87.5% indicating fairly good classification performance. Considering the other matrices evaluation in the figure, the system manages the challenges of COVID-19 and non-COVID-19 perfectly.

### 3.3. Dataset 3: COVID-19 and 20 of Non-COVID-19 Cases

**Figure 7:**
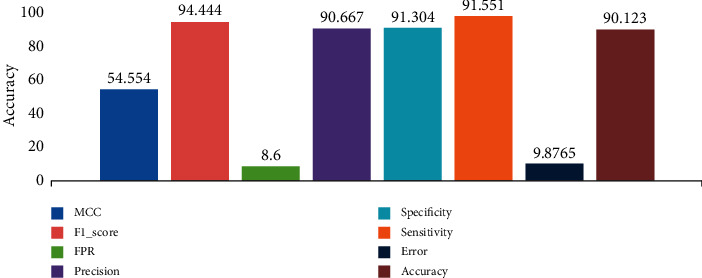
Overall values of COVID-19 with only 20 cases of Non-COVID-19 cases.

Dataset 3 shows a comparison between chest CT images of COVID-19 cases with non-COVID-19 cases (other lung diseases that have similar findings in chest CT scan). The researchers entered 349 CT images with different slices of confirmed cases of COVID-19 along with 396 non-COVID-19 images cases. The most important challenge facing COVID-19 and Non-COVID-19 cases is disease similarity; for this reason, the differentiation is very hard to measure between two cases. The total accuracy of the system with COVID-19 and non-COVID-19 is 90.123% indicating good performance as seen in Figure 7. The misclassification rate is 9.87% and the precision is 90.6% for the percentage of positive classes to the total number of positive prediction. The false-positive rate is 8.6% seen as an indicator of the proportion of the negative classes that were falsely classified.

### 3.4. Dataset 4: COVID-19 and All Non-COVID-19 Cases

Dataset 4 is a comparison between chest CT images of COVID-19 cases with non-COVID-19 cases (other lung diseases that have related chest CT scan observations as stated earlier). The researchers entered 349 CT images with different slices of confirmed cases of COVID-19 and 396 images of non-COVID-19 cases. According to the original dataset, the challenges in this case have increased by using the total number of non-COVID-19. This means that in the cases of other COVID-19 diseases, and by considering this difficulty, the system has achieved total accuracy of 85.14%. The rate of misclassification was 14.86% which reflected the number of metrics misclassified cases of both positive and negative classes. By considering the challenges and problems of the datasets, the performance is fairly good for this case.

### 3.5. Performance Comparison of COVID-19 Datasets

**Figure 8:**
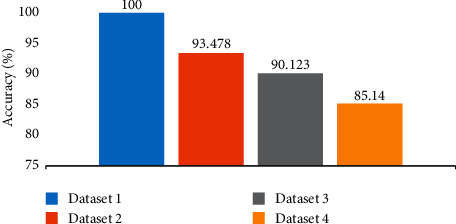
Total performance comparison of four datasets.

The evaluation comparison of four datasets that represent different challenges of differentiation and similarity is consistent. This is evident from dataset 1 of COVID-19 with normal cases which achieved high performance with 100% accuracy as shown in Figure 8. In dataset 2, COVID-19 with a limited number of non-COVID-19 with normal cases achieved an accuracy of 93.48%, as the level of challenges index becomes higher, the accuracy decreases. In dataset 3, COVID-19 with non-COVID-19 (other diseases) showed accuracy at 90.123%, reflecting the difficulty of distinguishing the similarity between the two cases. The most difficult cases were observed in dataset 4 containing countless COVID-19 (other diseases) which achieved 85.14% accuracy.

**Figure 9:**
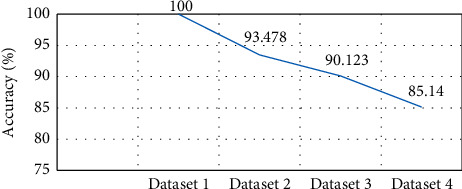
Datasets accuracy and challenges level representation.

The line of challenges for four datasets reflects the level of problems and this is clearly observed in the relationship between accuracy and level of challenges corresponding to the four datasets as seen in Figure 9. It can be stated that based on the result the accuracy will decrease as challenges increase in the datasets. This is considered the best protocol in order to diagnose the COVID-19 and help radiologists in their decision-making process.

The overall results achieved an excellent sensitivity of over 95%, at fewer than low evaluation in case of Golden Key Tool, and show an improvement of COVID-19 CT views. However, this facility can help respiratory specialists by giving a rapid triaging of radiological findings detection that increases the sensitivity of the diagnosis. The researcher's use of a Convolutional Neural Network on a chest CT case makes the recognition module more helpful when compared to other alternative approaches. A total of 800 images (349 COVID-19(+), 396 non-COVID-19 other diseases, and 55 normal which no-findings on chest CT) were used to develop the model. A fair result was obtained when the problems and the challenges of the dataset in this for binary and four groups were considered. The combination of individual systems with a new tool by new images view and by CNN classification process and detection illustrate a good impact on Radiology Order Entry Clinical Decision Support (CDS).

### 3.6. Challenges in the Datasets

The datasets were divided into four datasets that mean four protocols. The challenges in this paper present the protocol to help in the diagnoses of COVID-19 observed in CT findings in COVID-19 cases at different stages mainly related to pathogenesis. However, the consolidation superimposed on GGO as observed in the initial imaging presentation is found in a smaller number of cases, mainly among elderly people. With changes over time, very few cases have negative CT findings at the early stage. Advanced-phase disease is associated with a significantly increased frequency of CT pattern of COVID-19. There is much overlap of the CT-pattern of COVID-19 with other viral pneumonia and acute or chronic lung diseases. However, the advanced-phase disease makes the chest CT image more similar to non-COVID in some cases. Even though CNN is the best one to differentiate between image identities, there are small lines and time priority that can increase the false-negative rate, which may decrease the accuracy. In the case of vision evaluation, the quality difference between paper CT images and original CT images would not significantly harm the quality of the diagnosis as in the primary study but occurs in the case of CNN. If image enhancement is carried out before the convolution process to make the appearance of blurred or low resolution images better, the performance of a deep learning model can be enhanced. Missing data is a problem for deep learning networks because when large contiguous areas of images are missing, the network output deteriorates appreciably.

## 4. Conclusion

In the first study, the process is hardly new as a facility in the radiology department. But it continues to evolve in the face of newer technologies that can help and give more efficiency, improving the radiologist's performance and the accuracy in many cases as well as COVID-19 based on CT images. This tool provides impetus for early diagnosis and increases the sensitivity of the chest CT scan to distinguish the COVID-19 infection from other conditions. This study was assisted by specialists and experts who recommend that the appropriate test has the potential to make a difference in the field of radiology medicine. The second study focused on the diagnosis protocols by using different CNN classifications on chest CT cases of COVID-19 infection. These diagnosis protocols help the radiologists to make the right decisions. Moreover, the results can help respiratory specialists to study and observe the latest radiological findings which are based on patient management and may contribute to increasing diagnostic efficacy. Hence, this paper allows the usage of the new CT scan view and classification protocols based on challenges for other diseases.

## Figures and Tables

**Algorithm 1:**
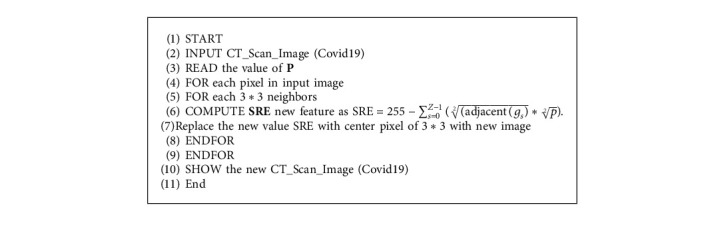
Pseudocode for square root equation.

**Table 1:** Network configuration. Details of VGG face CNN configuration where N is the number of classes.

Layer name	Input size	Filters	Padding, stride, filter size
Conv1	224 × 224	64	1, 1, 3
Conv2	224 × 224	64	1, 1, 3
Max Pooling (1)	224 × 224	—	0, 2, —
Conv3	112 × 112	128	1, 1, 3
Conv4	112 × 112	128	1, 1, 3
Max Pooling (2)	112 × 112	—	0, 2, —
Conv5	56 × 56	256	1, 1, 3
Conv6	56 × 56	256	1, 1, 3
Conv7	56 × 56	256	1, 1, 3
Max Pooling (3)	56 × 56	—	0, 2, —
Conv8	28 × 28	512	1, 1, 3
Conv9	28 × 28	512	1, 1, 3
Conv10	28 × 28	512	1, 1, 3
Max Pooling (4)	28 × 28	—	0, 2, —
Conv11	14 × 14	512	1, 1, 3
Conv12	14 × 28	512	1, 1, 3
Conv13	14 × 14	512	1, 1, 3
Max Pooling (5)	28 × 28	—	0, 2, —
Output	7 × 7		
Fully connected	4096		
Dropout	0.5		
Fully connected	4096		
Dropout	0.5		
Fully connected	N classes		
Softmax	N classes		

**Table 2:** The scale results illustrate a good assessment of the images view improvement by radiologists.

Good					
		Frequency	Percent	Valid percent	Cumulative percent
Valid	4.00	15	78.9	100.0	100.0
Missing	System	4	21.1		
Total		19	100.0		

**Table 3:** The scale results illustrate an excellent assessment of the images view improvement by radiologists.

Excellent					
		Frequency	Percent	Valid percent	Cumulative percent
Valid	5.00	18	94.7	100.0	100.0
Missing	System	1	5.3		
Total		19	100.0		

## Data Availability

The data used in this research are the CT views of COVID-19 patients, Wuhan, China. A senior radiologist at Tongji Hospital, Wuhan, China, has verified these data.
